# An induction of microRNA, miR-7 through estrogen treatment in breast carcinoma

**DOI:** 10.1186/1479-5876-10-S1-S2

**Published:** 2012-09-19

**Authors:** Mariko Masuda, Yasuhiro Miki, Shuko Hata, Kiyoshi Takagi, Minako Sakurai, Katsuhiko Ono, Koyu Suzuki, Yang Yang, Eriko Abe, Hisashi Hirakawa, Takanori Ishida, Takashi Suzuki, Noriaki Ohuchi, Hironobu Sasano

**Affiliations:** 1 Department of Pathology, Tohoku University Graduate School of Medicine, Sendai, Japan; 2 Department of Pathology, St. Luke’s International Hospital, Tokyo, Japan; 3 Department of Surgery, Tohoku Kosai Hospital, Sendai, Japan; 4 Department of Surgical Oncology, Tohoku University Graduate School of Medicine, Sendai, Japan

## Abstract

**Background:**

Estrogen plays an important role in the development of estrogen-dependent breast carcinoma. Recently, several studies demonstrated a possible involvement of several micro RNAs (miRNAs) in the development of resistance to endocrine therapy in breast cancer patients, but the correlation between estrogen actions and miRNA expression in breast carcinoma still remains largely unknown. Therefore, in this study, we examined the *in vitro* effects of estrogen upon miRNA expression profiles in breast carcinoma.

**Methods:**

We first screened the miRNA expression profiles induced by 17β-Estradiol (E2) using RT^2^ miRNA PCR Array in the ER-positive breast carcinoma cell line MCF-7. We identified miR-7 as the important miRNA associated with estrogen actions in these cells and further examined the changes of estrogen-dependent EGFR expression by miR-7 in ER-positive or -negative breast carcinoma cell lines including MCF-7. We also evaluated the correlation between miR-7 and EGFR expression in breast carcinoma cells derived from 21 patients using laser capture microdissection combined with quantitative reverse transcriptase-PCR.

**Results:**

Seventeen miRNAs were significantly induced by E2 treatment in the MCF-7 cell line. Among 17 miRNAs induced by estradiol treatment, only miR-7 expression was significantly decreased by subsequent ICI treatment. The expression of miR-7 was up-regulated 2.94-fold by E2 treatment. miR-7 was reported to suppress epidermal growth factor receptor (EGFR) expression in several human malignancies. Transfection of miR-7 significantly suppressed EGFR mRNA levels in MCF-7 cells. Depletion of E2 from cell culture media also increased the expression level of EGFR mRNA in MCF-7 and T-47D cells but not in ER-negative, MDA-MB-231 and SK-BR-3 cells. We also evaluated the status of miR-7 in breast carcinoma tissues, but the correlation between the status of miR-7 and EGFR in carcinoma cells isolated by laser capture microscopy was not detected.

**Conclusions:**

These results suggest that miR-7 may play a role in the development of resistance to endocrine therapy in breast cancer patients through regulating EGFR expression of carcinoma cells.

## Background

Breast cancer is one of the leading causes of cancer deaths in women. Estrogen stimulates breast cancer cell proliferation by binding to the estrogen receptor (ER) and the estrogen-ER complex interacts directly with estrogen response element (ERE), located in the promoter regions of the target genes to either activate or repress gene expression. Aromatase inhibitors are currently employed in clinical practice for treatment of postmenopausal ER-positive breast cancer patients, but the acquired endocrine resistance has become one of the major problems in the clinical management of these patients. Several studies have demonstrated that activation of other cell proliferation pathways involving epidermal growth factor receptor (EGFR) and/or insulin-like growth factor 1 receptor (IGF-1R) in breast cancer cells can be at least partly responsible for the subsequent development of resistance to endocrine therapy [1,2]. The molecular mechanisms of conversion from ER signals to other proliferative signals in breast cancer cells however remain largely unknown.

MicroRNAs (miRNAs) are small (20-24 nucleotides), non-coding RNA gene products, which post-transcriptionally modulate gene expression by negatively regulating the stability or transcriptional efficiency of their target mRNAs. miRNAs have demonstrated in playing key regulating roles in various cellular processes, including cell cycle, differentiation, development and metabolism [3-5]. Aberrant expressions of miRNAs have been reported to be associated with malignant phenotypes in various human tissues, and some miRNAs might also function as tumor suppressor genes or oncogenes [6]. Several miRNAs are specifically expressed in breast cancer tissues [7]. In particular, miR-206 was reported to be associated with estrogen signals by targeting *ESR1* (ERα) of breast carcinoma cell lines [8], and miR-221/222 could regulate estrogenic signals through ERα, resulting in the development of anti-estrogen resistant breast cancer [9,10]. Estradiol (E2)-regulated miRNAs were also expressed in breast carcinoma cell lines. Maillot et al. [11] reported that eight miRNAs were repressed by E2 and might be involved in the tumor response to hormonal therapy, but the expression of E2/ER-inducible miRNAs were different between published reports [8-11]. In general, miRNAs were demonstrated to be regulated by estrogen and their functions still remain unclear.

Therefore, in this study, we first screened miRNAs induced by E2 treatment using RT-PCR array in the ER-positive breast cancer cell line MCF-7, among human cancer-related miRNA expression profiles. We then examined the possible correlation between EGFR mRNA expression and ER/E2-inducible miRNA which was screened out from the above analysis using breast carcinoma cell lines and tissues.

## Methods

### Chemicals

E2 was commercially obtained from Wako Pure Chemical Industries (Osaka, Japan) and ICI 182, 780 (ICI) from Tocris Cookson (Ellisville, MO, USA). Both chemicals were dissolved in pure ethanol.

### Breast cancer cases

A total of 41 cases of breast cancer surgical specimens were evaluated in this study. Twenty-one cases of invasive ductal carcinoma of the breast were obtained from Tohoku Kosai Hospital (Sendai, Japan), 6 cases from St. Luke’s International Hospital (Tokyo, Japan) and 14 cases from Tohoku University Hospital (Sendai, Japan). The research protocol for this study was approved by the Ethics Committees of Tohoku Kosai Hospital (No. H17. 8. 5), St. Luke’s International Hospital (No. 10-030), and Tohoku University, School of Medicine (No. 2009-203). Clinicopathological features of these cases were summarized in Table 1. For laser capture microscopy evaluation, a total of 20 cases of breast carcinoma frozen tissues embedded in OCT compound and 21 cases of 10% formalin-fixed and paraffin embedded (FFPE) tissue specimens were evaluated.

**Table 1:** Summary of clinicopathological characteristics of the patients examined in this study

Characteristics	Number of patients, n (%)	
	
	Frozen tissues (n=20)	FFPE tissues (n=21)
Age (median, range)	58 (41-76) years	55 (31-86) years
Menopausal status		
Pre-menopausal	5 (25%)	4 (19%)
Post-menopausal	15 (75%)	17 (81%)
Type		
IDC	19 (95%)	21 (100%)
DCIS	1 (5%)	0 (0%)
ER status		
Positive	16 (80%)	14 (67%)
Negative	4 (20%)	7 (33%)
PR status		
Positive	14 (70%)	12 (57%)
Negative	6 (30%)	9 (43%)
HER2 score		
0, 1+, 2+	19 (95%)	4 (19%)
3+	1 (5%)	17 (81%)

FFPE, 10% formalin-fixed and paraffin embedded; IDC, invasive ductal carcinoma; DCIS, ductal carcinoma *in situ*; ER, estrogen receptor; PR, progesterone receptor.

### Breast carcinoma cell lines and culture conditions

The human breast carcinoma cell lines MCF-7 and SK-BR-3 were provided by the Cell Resource Center for Biomedical Research, Tohoku University (Sendai, Japan), and MDA-MB-231 and T-47D were commercially obtained from the American Type Cell Culture (Manassas, VA). All the cell lines were cultured in RPMI 1640 (Sigma-Aldrich, St Louis, MO) and supplemented with 10% fetal bovine serum (FBS; Nichirei Biosciences, Tokyo, Japan).

### Analysis of miRNA in breast carcinoma cell lines

Total RNA was carefully extracted from breast carcinoma cell lines using the TRIzol method (Invitrogen, Carlsbad, CA). cDNA for mRNA-PCR was synthesized using a QuantiTect Reverse Transcription kit (QIAGEN, Hilden, Germany), and cDNA for miRNA-PCR was synthesized using a RT^2^ miRNA First Strand Kit (QIAGEN).

### Analysis of miRNAs in human breast cancer cases using laser capture microdissection (LCM)

The tissue sections of 8 μm were used for LCM/qPCR. LCM was performed using mmi CellCut (MMI Molecular Machines and Industries, Flughofstrasse, Glattbrugg, Switzerland). Approximately 5,000 cells were laser transferred from carcinoma and the total RNA was extracted using RNeasy Micro Kit (QIAGEN). cDNA was synthesized using a QuantiTect Reverse Transcription kit (QIAGEN). FFPE sections (8 μm) of breast carcinoma were also used for evaluation of miRNA in these 21 cases. The tissue sections were deparaffinized using heating slides set at 60°C for 3 hours. LCM was performed using mmi CellCut as in the analysis using frozen tissue sections. miRNA was extracted using PureLink miRNA Isolation Kit (Invitrogen). cDNA was also synthesized using a RT^2^ miRNA First Strand Kit (QIAGEN).

### miRNA PCR array

MCF-7 treated with E2 or combination of E2 and ICI were analyzed for the expression of a panel of 88 cancer-related miRNAs using Human Cancer RT^2^ miRNA PCR Array (QIAGEN). PCR was performed in ABI7500 Real-Time PCR System (Applied Biosystems, Foster city, CA) at the Biomedical Research Core of Tohoku University (Sendai, Japan). Data analyses were performed with the web-based software package for the RT2 Profiler PCR Array Data Analysis (http://www.Sabiosciences.com/pcr/arrayanalysis.php).

### Real time RT-PCR

Real time RT-PCR was carried out using the LightCycler System (Roche Diagnosis, Mannheim, Germany) and FastStart DNA Master SYBR Green I (Roche Diagnostics). The primer sequences used in this study were as follows: RPL13A (NM_012423) forward 5'-CCT GGA GGA GAA GAG GAA AG-3'; RPL13A reverse 5'-TTG AGG ACC TCT GTG TAT TT-3'; ESR1 (ER, NM_000125) forward 5'-AGA CAC TTT GAT CCA CCT GA-3'; 5'-CAA GGA ATG CGA TGA AGT AG-3'; EGFR (NM_005228) forward 5'-CAG CTA TGA GAT GGA GGA AG-3'; reverse 5'-CGT AGC ATT TAT GGA GAG TGA G-3'. Real-time PCR was performed under the following conditions: ER, 10 min initial hold at 95°C, 40 cycles of 95°C for 10 s, 62°C for 10 s and 72°C for 15 s, cooling to 40°C and EGFR, 10 min initial hold at 95°C, 40 cycles of 95°C for 10 s, 67°C for 10 s and 72°C for 10 s, cooling to 40°C. cDNAs of known concentrations for target genes and the housekeeping gene, RPL13A were used to generate standard curves for real-time quantitative PCR in order to determine the quantity of target cDNA transcripts. The mRNA level in each case is represented as a ratio of RPL13A (%).

### Quantitative RT-PCR of miR-7

RT^2^ miRNA PCR Assay was carried out using the ABI7500 Real-Time PCR System and RT2 SYBR Green/ROX qPCR Master Mix (QIAGEN). The primers used in this study were hsa-miR-7 (MIMAT0000252) and U6 (NR-002752.1). Real-time PCR was performed under the following conditions; 10 min initial hold at 95°C, 40 cycles of 95°C for 15 s, 60°C for 40 s and 72°C for 30 s. U6 was used as a housekeeping gene, and expression levels of a miRNA of interest were normalized to U6 level.

### Search for putative targets of miR-7

We used the TargetScan (http://www.targetscan.org/), Microcosm Targets (http://www.ebi.ac.uk/enright-srv/microcosm/htdocs/targets/v5/) and microRNA.org. (http://www.microrna.org/microrna/home.do) to compute miR-7 target predictions. Almost all known miRNA binding site are located in the 3’ untranslated region of target mRNA and target databases contained mRNA conserved binding site of miRNA.

### miR-7 transfection

MCF-7 cells were plated in 6-well plates on phenol red-free RPMI 1640 supplemented with 10% dextran-coated charcoal FBS (DCC-FBS). Transient transfections were performed with 10 nM miR-7 (has-miR-7; 5’-UGGAAGACUAGUGAUUUUGUUGU-3’) using G-fectin (Genolution Pharmaceutical, Seoul, Korea). Scramble RNA (Genolution Pharmaceutical) was employed for negative control. The culture medium was exchanged with miR-7 on three days after the first transfection. EGFR mRNA levels were evaluated using real time PCR described above on fifth day from the first transfection.

### Effects of E2 depletion on cultured cells

For estrogen depletion studies, cells were cultured in phenol-red-free RPMI 1640 (Sigma-Aldrich) supplemented with 10% DCC-FBS. Estrogen and androgen concentrations in this medium were below the detection limits measured by LC-MS/MS. After five days, EGFR mRNA levels were evaluated using the real time PCR described above. MiR-7 expression levels in MCF-7 cultivated with normal FBS and DCC-FBS were also evaluated using the quantitative RT-PCR method described above.

### Statistical analysis

Statistical analysis was performed using StatView 5.0J software (SAS Institute, Cary, NC, USA). qRT-PCR data were analyzed using analysis of variance followed by the post-hoc Bonferroni/Dunnet multiple comparison test. A p-value < 0.05 was considered to be statistically significant.

## Results

### miRNA expression profiles in MCF-7 cell treated with E2

**Figure 1:**
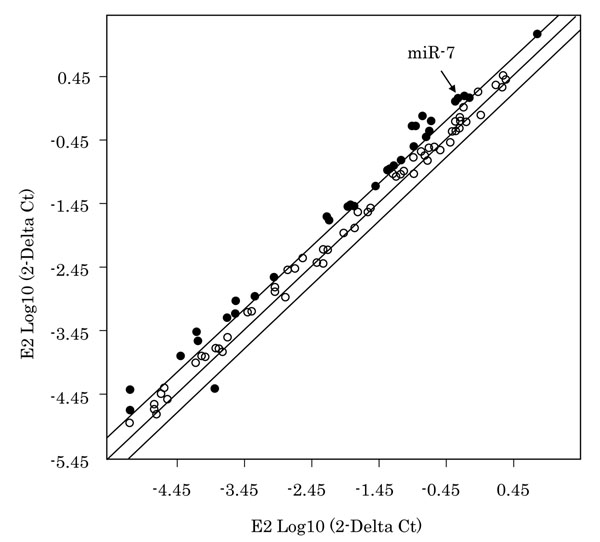
Results of the status of miRNA expression in MCF-7 cell line treated with E2. Scattered plot analysis of miRNA PCR array in MCF-7 treated for 24 hours. Solid lines, y = x with lines indicating two fold increment or decrement of expression levels.

We examined the miRNA profiling in MCF-7 cells treated with 10 pM E2 or combination of 1 μM ICI and 10 pM E2 in order to identify the E2-ER regulated miRNAs. Those with expression ratios above 2.0-fold compared to control cells following 24 hours were summarized in Fig. 1 and Table 2. 17 miRNAs were significantly up-regulated following E2 treatment (Fig. 1, Table 2). Among these 17 miRNAs induced by E2, only mir-7 was significantly decreased in its expression by the treatment of ICI compound (Table2). Therefore, in this study, we focused on expression of miR-7 and further examined biological feature of miR-7 as an estrogen inducible miRNA in breast carcinoma cells.

**Table 2:** Summary of miRNAs up-regulated by E2 in MCF7 cell line

Gene name	E2		E2+ICI
	
	Fold change*	*p*-value	Fold change**
miR-301a	5.29	0.037	-2.44
miR-20a	5.25	0.007	-1.09
miR-34c-5p	3.75	0.030	1.13
miR-149	3.53	0.017	-1.28
miR-17	3.36	0.001	-1.37
miR-206	3.35	0.003	nd
miR-127-5p	3.27	0.001	nd
miR-25	2.94	0.031	-1.70
miR-7	2.94	0.001	-2.77 (s)
miR-191	2.90	0.046	-1.33
miR-27b	2.65	0.001	-1.02
miR-124	2.65	0.022	7.21
miR-181d	2.40	0.008	1.19
miR-148a	2.31	0.031	-1.06
miR-29a	2.29	0.015	1.10
miR-21	2.05	0.008	1.42
miR-210	2.01	0.032	-1.81

miRNAs of > 2.0 fold up-regulated in MCF-7 cells are listed.

E2, 10pM estradiol; ICI; 1μM ICI182,780; nd, under detectable limit; s, significant (p=0.008); *, >2- fold of vehicle control; **, fold change of E2.

### Effects of miR-7 on EGFR mRNA expression in MCF-7

**Figure 2:**
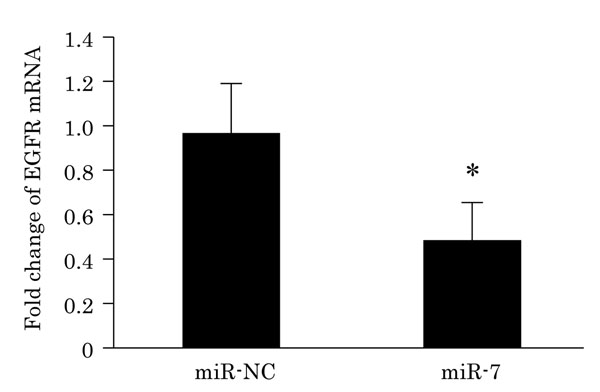
Effects of miR-7 upon EGFR mRNA abundance in MCF-7 cell line. EGFR mRNA levels in MCF-7 transfected with miR-7 for five days. *, Significantly different from miR-NC. p < 0.05. miR-NC, transfection with scramble RNA.

Analysis of potential target genes of miR-7 was evaluated using TargetScan, Microcosm Targets, and microRNA.org target prediction. Results indicated EGFR as one of the target genes of miR-7. Therefore, in this study, we focused on the correlation between EGFR mRNA and miR-7 expression. Results of miR-7 transfection assay demonstrated that EGFR mRNA was significantly decreased in MCF-7 transfected with miR-7 compared to those transfected with control scramble RNA (Fig. 2). Other EGFR family such as HER2, HER3, and HER4 were not potential target gene of miR-7 in three target prediction algorisms described above.

### Effects of E2 on EGFR mRNA in breast carcinoma cells

**Figure 3:**
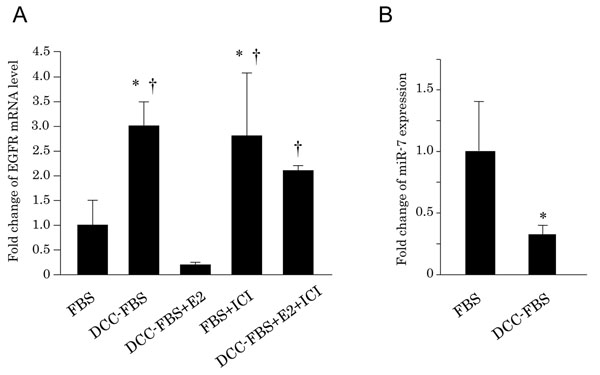
(A) Effects of E2 upon EGFR mRNA in MCF-7. EGFR mRNA levels were evaluated using quantitative RT-PCR after 5 days of culture in each condition. (B) Alterations of miR-7 expression by depletion of E2 in MCF-7. FBS, Medium supplemented with 10% FBS; DCC-FBS, Medium supplemented with 10% DCC-FBS; E2, treatment of 10 nM E2; ICI, treatment of 1 μM ICI. *, Significantly different from FBS; †, Significantly different from E2.

The amounts of EGFR mRNA were significantly increased by the removal of E2 from the culture medium in MCF-7 cells. This EGFR induction was significantly decreased by an addition of 10 nM E2, but the effect was significantly decreased by 1μM ICI treatment in the E2-depleted MCF-7 cells (Fig. 3A). The ICI treatment significantly increased the levels of EGFR mRNA expression in MCF-7 cells without E2 depletion (Fig. 3A). The miR-7 expression level was significantly decreased by the depletion of E2 in MCF-7 cells (Fig. 3B).

**Figure 4:**
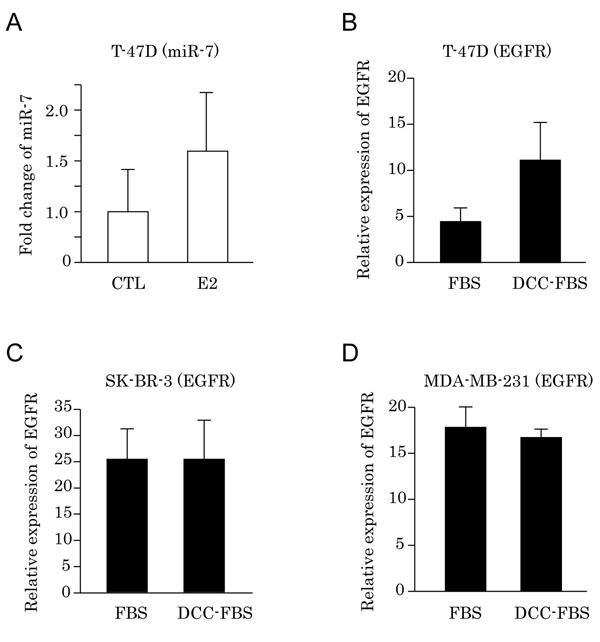
(A) Alterations of miR-7 expression by depletion of E2 in T-47D. Levels of miR-7 expression were normalized using U6 probe. (B-D): Alterations of EGFR mRNA expression by depletion of E2 in breast carcinoma cells. Values for EGFR mRNA were depicted as percent expression to RPL13A by quantitative RT-PCR. (B) Expression of EGFR mRNA in ER-positive T-47D cells cultured in medium supplemented with 10% FBS or DCC-FBS. (C, D): Expression of EGFR mRNA in ER-negative SK-BR-3 (B) and MDA-MB-231 (C) cells cultured in medium supplemented with 10% FBS or DCC-FBS.

In T-47D cells, the expression levels of miR-7 (Fig. 4A) and EGFR mRNA (Fig. 4B) were increased by E2 (10nM) treatment and depletion of E2, respectively. However, the changes did not reach statistical significance. E2 depletion in cell culture medium did not influence the levels of EGFR mRNA in ER-negative MDA-MB-231 and SK-BR-3 cell lines (Fig. 4C, D).

### Expression of miR-7 in breast cancer tissues

**Figure 5:**
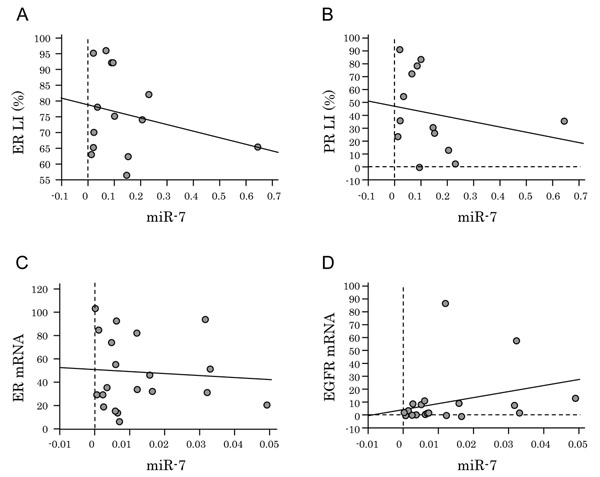
MiR-7 expression in breast cancer tissues. Levels of miR-7 expression were normalized using U6 probe. Correlation between miR-7 and (A) ER or (B) PR LIs (%) in fourteen ER-positive FFPE breast carcinoma tissues. Correlation between miR-7 and (C) ER or (D) EGFR mRNA in twenty fresh frozen breast carcinoma tissues.

MiR-7 was detected in both fresh frozen and FFPE breast carcinoma tissues. No significant correlations were detected between the levels of miR-7 expression and ER (Fig. 5A) or PR (Fig. 5B) LIs in 21 ER-positive breast carcinoma cases. There were no significant correlations between miR-7 and ER (Fig. 5C) or EGFR (Fig. 5D) mRNA detected in carcinoma cells isolated by LCM from 20 ER-positive fresh frozen breast carcinoma tissues in this study.

## Discussion

Many investigators have reported the regulation of miRNAs in estrogen actions in breast carcinoma cells [7-11]. However, these reported results are not consistent. Maillot et al. [11] reported that eight miRNAs (miR-181a, miR-26a, miR-181b, miR-26b, miR-200c, miR-21, miR-23b and miR-27b) were down-regulated by E2 in MCF-7 cells but these miRNAs were not identified among E2-repressed miRNAs in our present study. We identified miR-21 and miR-27b as E2-induced miRNAs and Bhat-Naksharti et al. [12] also reported an increment of miR-21 by E2 treatment. These discrepancies regarding the correlation between estrogen actions and miRNA profiles in breast carcinoma cells among reported studies may be due to the different cell culture conditions including the treatment time and the dose of E2. It was reported that E2-induced increment of miRNAs started to be detected only after 18 hours of E2 treatment because of the stability of the mature miRNA [11]. Therefore, we evaluated alterations of miRNA by E2 as reported by Maillot et al [11]. In our present study, among 17 miRNAs induced by E2 treatment, ICI treatment significantly decreased the expression of miR-7 alone. The miRNAs induced by estrogen treatment may be inhibited by the co-treatment with ER antagonist, but several studies demonstrated that some miRNAs were induced by ICI treatment alone in endometrial cells and breast carcinoma cells including MCF-7 cells [13-15]. Further studies including specific inhibition assays using siRNA of ERα are required to clarify an induction of miRNAs through the ERα.

MiR-7 belongs to intronic miRNA and is present in an intron of *hnRNP K* (heterogeneous nuclear ribonucleic protein K) in both insects and mammals [16,17]. The amounts of hnRNP K expression were also reported to be relatively higher in ER^+^/PR^+^ primary breast tumors compared to ER^–^/PR^–^ tissues [18]. In addition, ERE is located in the 5’ flanking region of the hnRNP K gene [18]. These results suggested that an up-regulation of miR-7 by E2 was due to the splicing of hnRNP K, which was also increased by E2 treatment in MCF-7 cells [18]. In this study, we also examined the expression of miR-7 in T-47D cells. The miR-7 expression was increased by E2 treatment in T-47D cells but the change did not reach statistical significance (p=0.0508). ERβ expression was detected predominantly in T-47D cells but not in MCF-7 cells (data not presented). Therefore, the induction of miR-7 by E2 treatment through the hnRNP K is considered to be influenced by the expression patterns of ER subtypes in the cells.

In our present study, we focused on the change in EGFR expression because miR-7 was reported to inhibit EGFR expression and subsequently suppress cell proliferation in several human carcinoma cell lines [19-21]. E2-depletion from culture media of MCF-7 cells suppressed miR-7 expression and increased EGFR expression. In addition, EGFR mRNA expression was also suppressed by miR-7 transfection in MCF-7 cells. These results all indicated that the signals regulated E2-induced miR-7 expression through EGFR, and the inhibition or suppression of ER-mediated signaling suppressed miR-7 and subsequently increased EGFR mRNA expression in ER-positive breast carcinoma cells. The EGFR overexpression by E2 depletion may be considered within the spectrum of the survival mechanisms to avoid cell death in carcinoma cells. This finding may also account partly for an emergence of alternative proliferative pathways for the survival of carcinoma cells under endocrine treatment. However, the detailed mechanisms regarding this ‘switching theory’ in breast carcinoma cells treated with endocrine therapy remain largely unknown. The results of our present study also suggest that miR-7 may be involved in these cell survival mechanisms of breast carcinoma cells under estrogen depletion, but further investigations are warranted.

An overexpression of growth factor receptors such as EGFR has been also reported as one of the mechanisms of resistance to endocrine therapy in ER-positive breast cancer patients [1,2,22]. Many clinical trials studied the efficacy of adding an inhibitor of growth factor receptors such as gefitinib to endocrine therapy such as tamoxifen or aromatase inhibitors [23-26]. MiR-7 was also reported to regulate *IGF-1R* expression in tongue squamous cell carcinoma cells [27]. In breast carcinoma cells, an interaction between ERα and IGF-1R has been recognized to enhance proliferative activity [28-30]. In addition, E2 treatment was also known to increase the expression of IGF-1R protein in MCF-7 cells [31]. In our present study, IGF-1R mRNA was relatively low in MCF-7 cells cultivated in E2-depleted condition (data not presented). Therefore, miR-7-mediated actions are considered to be related to EGFR than IGF-1R in MCF-7 cells. However, the correlation between miR-7 and ER or EGFR was not detected in our *in vitro* analysis of the tumor samples of breast cancer patients, but none of them received endocrine therapy before surgery. Therefore, further examinations including the alterations of the miR-7 expression in those who received neoadjuvant endocrine therapy are required to clarify the roles of miR-7 in ER and EGFR signaling.

## Conclusions

Aromatase inhibitors are commonly used as hormone therapy in postmenopausal estrogen-sensitive breast cancer patients. It is true that both plasma and intratumoral estrogen concentration was decreased by the treatment of aromatase inhibitor in postmenopausal ER-positive patients [32]. Acquired resistance to aromatase inhibitor has become one of the major problems in the clinical management of these patients. It is possible that the development of endocrine resistance is attributed to the up-regulation of EGFR caused by estrogen depletion which represents an attempt to rescue cell growth by switching to alternative pathway. The results of our study suggest that miR-7 may play central roles in the development of resistance to endocrine therapy in breast cancer patients through regulating EGFR expression of cancer cells.

## Abbreviations

DCC-FBS: 10% dextran-coated charcoal fetal bovine serum; E2: Estradiol; EGFR: epidermal growth factor receptor; ER: estrogen receptor; ERE: estrogen response element; FBS: fetal bovine serum; FFPE: 10% formalin-fixed and paraffin embedded tissue; hnRNP K: heterogeneous nuclear ribonucleic protein K; ICI: ICI 182, 780; IGF-1R: insulin-like growth factor 1 receptor; LCM: laser capture microdissection; miRNA: microRNA; MRP1: multidrug resistance protein 1; PCR: polymerase chain reaction; RT-PCR: reverse-transcriptase polymerase chain reaction.

## Competing interests

YM and HS received the educational research grant from Pfizer Japan Inc. The other authors have no conflict of interest.

## Authors' contributions

YM conceived and designed the study. MM performed most of the experiments and drafted the manuscript. KS, YY, HH, TI, and NO collected and stored all the samples. SH, KT, and KO contributed in miRNA array analysis, immunohistochemistry, and LCM respectively. YM, HS, MS and EA performed experiments for revised manuscript. TS and YM performed data analysis and histopathological correlations. HS supervised all experiments. YM and HS edited the manuscript. All authors read and approved the final manuscript.
